# The diagnosis of food allergy: protocol for a systematic review

**DOI:** 10.1186/2045-7022-3-18

**Published:** 2013-06-07

**Authors:** Karla Soares-Weiser, Sukhmeet S Panesar, Tamara Rader, Yemisi Takwoingi, Thomas Werfel, Antonella Muraro, Karin Hoffmann-Sommergruber, Graham Roberts, Aziz Sheikh

**Affiliations:** 1 Enhance Reviews Ltd, Central Office, Cobweb Buildings, The Lane, Lyford, Wantage, OX12 0EE, UK; 2 University of Edinburgh, Teviot Place, Edinburgh, EH8 9AG, UK; 3 University of Ottawa, 75 Laurier Avenue East, Ottawa, ON K1N 6N5, Canada; 4 University of Birmingham, Birmingham, UK; 5 Hanover Medical School, Carl-Neuberg-Straße 1, Hanover, 30625, Germany; 6 Padua General University Hospital, Via Giustiniani 3, Padua, 35128, Italy; 7 Medical University of Vienna, Spitalgasse 23, Vienna, 1090, Austria; 8 Faculty of Medicine, University of Southampton, Southampton, SO171BJ, UK; 9 University of Edinburgh, Teviot Place, Edinburgh, EH8 9AG, UK; 10 Centre for Population Health Sciences, The University of Edinburgh Medical School, Doorway 3, Teviot Place, Edinburgh, EH8 9AG, UK

**Keywords:** Food allergy, IgE-mediated, Diagnosis, Diagnostic tests

## Abstract

**Background:**

The literature on diagnostic tests for food allergy currently lacks clear consensus regarding the accuracy and safety of different investigative approaches. The European Academy of Allergy and Clinical Immunology is in the process of developing its *Guideline for Food Allergy and Anaphylaxis*, and this systematic review is one of seven inter-linked evidence syntheses that are being undertaken in order to provide a state-of-the-art synopsis of the current evidence base in relation to epidemiology, prevention, diagnosis and clinical management, and impact on quality of life, which will be used to inform the formulation of clinical recommendations. The aim of this systematic review will be to assess the diagnostic accuracy of tests aimed at supporting the clinical diagnosis of IgE-mediated food allergy.

**Methods:**

The following databases from inception to September 30, 2012 will be searched for studies of diagnostic tests: Cochrane Library (Wiley&Sons); MEDLINE (OVID); Embase (OVID); CINAHL (Ebscohost); ISI Web of Science (Thomson Web of Knowledge); TRIP Database (web http://www.tripdatabase.com); and Clinicaltrials.gov (NIH web). These database searches will be supplemented by contacting an international panel of experts. Studies evaluating APT, SPT, specific-IgE, and component specific-IgE in participants of any age with suspected food allergy will be included. The reference standard will be DBPCFC in at least 50% of the participants. Studies will be quality assessed by using the QUADAS-2 instrument. We will report summary statistics such as sensitivity, specificity, and/or likelihood ratios. We will use the hierarchical summary ROC (HSROC) model to summarize the accuracy of each test and to compare the accuracy of two or more tests.

**Discussion:**

Decisions on which tests to use need to be guided by availability of tests, populations being cared for, risks, financial considerations and test properties**.** This review will examine papers from around the world, covering children and adults with suspected food allergy in varying populations and concentrated on four type of tests: APT, SPT, specific-IgEs, and component specific-IgEs.

## Background

The umbrella term ‘food hypersensitivity’ can be used to describe any ‘adverse reaction to food’ [1]. The term ‘food allergy’ refers to the subgroup of food-triggered reactions in which immunologic mechanisms have been implicated, whether IgE-mediated, non-IgE-mediated, or involving a combination of IgE- and non-IgE-mediated etiologies [2]. All other reactions to food that were in the past sometimes referred to as ‘food intolerance’ constitute non-allergic food hypersensitivity reactions and are out of the focus of this enquiry. Coeliac disease is an important non-IgE mediated condition but as it has distinct symptoms and prognosis different from atopic conditions it will be excluded from this review [3].

Allergic sensitisation to a specific food does not always lead to clinical reactions. Consequently, serological tests for food-specific IgE or the determination of positive skin prick test results are in of themselves insufficient to establish the diagnosis of IgE-mediated food allergy. Rather, there must also be evidence of the clinical expression of disease. These IgE-mediated reactions can have a number of clinical expressions, including angioedema, urticaria, atopic eczema/dermatitis, oral allergy syndrome and anaphylaxis. Non-IgE-mediated immunologic reactions result from activation of other immunologic pathways (e.g. T-cell mediated) and can manifest as atopic eczema/dermatitis, gastro-esophageal reflux disease, food protein-induced enterocolitis, proctocolitis, and enteropathy syndromes. The contemporary definition of food allergy thus includes several clinical entities with different pathophysiologies (see Table 1 below) resulting from exposure to different foods [3].

**Table 1:** Pathologies with respective disorders seen in food allergy

***Pathology***	***Disorder***
IgE-mediated (acute-onset)	• Acute urticaria (weals, angioedema or both)
	• Contact urticaria
	• Atopic eczema/dermatitis
	• Anaphylaxis
	• Food-associated, exercise-induced anaphylaxis
	• Oral allergy syndrome (pollen-associated food allergy syndrome)
	• Immediate gastrointestinal hypersensitivity
Cell-mediated (delayed onset/chronic)	• Atopic eczema/dermatitis
	• Food protein-induced enterocolitis syndrome
	• Food protein-induced allergic proctocolitis
	• Allergic contact dermatitis
	• Heiner syndrome
Combined IgE and cell-mediated (delayed onset/chronic)	• Atopic eczema/dermatitis
	• Eosinophilic oesophagitis
	• Eosinophilic gastroenteritis

The first and most important step in the diagnosis of food allergy involves a full clinical history and clinical examination. Numerous diagnostic tests have been proposed as useful adjuncts in those with a suggestive clinical history. The most commonly studied are skin prick testing (SPT), serum food-specific IgE determinations and atopy patch testing (APT), although APT is not in widespread clinical use. The double-blind placebo controlled food challenge (DBPCFC) is usually considered the gold standard diagnostic test. Food challenge tests are however time-consuming and resource-intensive, particularly if undertaken in a double-blind manner. Other tests that have been investigated for diagnosing food allergy include histamine, tryptase and chymase assays [2].

The literature on diagnostic tests for food allergy currently lacks clear consensus regarding the accuracy and safety of different investigative approaches. The European Academy of Allergy and Clinical Immunology (EAACI) is in the process of developing the *EAACI Guideline for Food Allergy and Anaphylaxis*, and this systematic review is one of seven inter-linked evidence syntheses that are being undertaken in order to provide a state-of-the-art synopsis of the current evidence base in relation to epidemiology, prevention, diagnosis and clinical management, and impact on quality of life, which will be used to inform the formulation of clinical recommendations.

### Aims

The aim of this systematic review will be to assess the diagnostic accuracy of tests aimed at supporting the clinical diagnosis of IgE-mediated food allergy.

## Methods

### Search strategy

**Figure 1:**
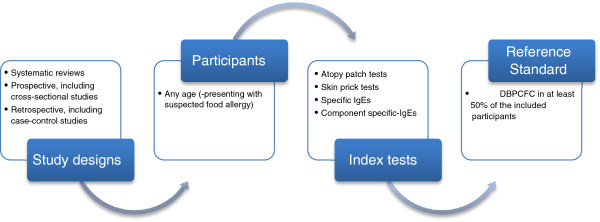
Conceptualisation of systematic review of diagnostic tests for food allergy.

A highly sensitive search strategy has been developed and validated study design filters will be applied to retrieve all articles pertaining to the diagnosis of food allergy from electronic bibliographic databases. We have conceptualised the search to incorporate three elements as shown in Figure 1: Conceptualisation of systematic review of diagnostic tests for food allergy.

To retrieve diagnostic studies, we will use the diagnosis filter developed at McMaster University Health Information Research Unit (HIRU) [4].

We will search the following databases:

•Cochrane Library, including:

○ Cochrane Database of Systematic Reviews     (CDSR)

○ Database of Reviews of Effectiveness (DARE)

○ CENTRAL (Trials)

○ Methods Studies

○ Health Technology Assessments (HTA)

○ Economic Evaluations Database (EED)

•MEDLINE (OVID)

•Embase (OVID)

•CINAHL (Ebscohost)

•ISI Web of Science (Thomson Web of Knowledge)

•TRIP Database (www.tripdatabase.com)

•Clinicaltrials.gov (NIH web)

The search strategy has been devised on OVID MEDLINE and then adapted for the other databases (see Additional file 1: for full search strategies). In all cases, the databases will be searched from inception to 30 September 2012. All references will be imported into an EndNote Library and tagged with the name of the database. Additional references will be located through searching the references cited by the identified studies, and unpublished work and research in progress will be identified through discussion with experts in the field. We will invite experts who are active in the field from a range of disciplines and locations to comment on our search strategy and the list of included studies. There are no language restrictions and, where possible, all literature will be translated.

### Inclusion criteria

•Study design:

○ The studies of diagnostic tests must have     sufficient data to calculate sensitivity, specificity,     and negative and positive predictive values.

○ Studies with prospective, cross-sectional or     retrospective design will be included. The    studies must have a defined study population,     and specify the recruitment method, either     consecutive or random sampling of participants.    Where the recruitment method is not clearly     reported, we will include such studies and assess     their influence on test performance through     undertaking sensitivity analyses.

•Types of participants:

○ Any age (children and adults) presenting    with suspected food allergy.

•Target conditions:

○ Studies examining the diagnostic accuracy of     tests for IgE-mediated food allergies (see Table 1),     against any type of food will be included.

•Index tests: We will document the tests that have been studied, but will focus in our analysis on:

▪ APT

▪ SPT

▪ Specific-IgE

▪ Component specific-IgE

○ For IgE-mediated food allergy:

•Reference standard:

○ The reference standard will be the DBPCFC,     which is considered the gold standard for     diagnosing food allergy. Where studies used    either theDBPCFC or an open food challenge     (OFC), these will be included if more than    50% of the participants received DBPCFC.

### Exclusion criteria

•Study design:

○ Reviews, discussion papers, non-research letters     and editorials

○ Qualitative studies

○ Case studies, case series

○ Animal studies

•Types of participants:

○ Studies will be excluded if the patients were     selected based on having a positive food allergy     test result (index test or reference standard).

•Analysis:

○ We will conduct a per patient analyses, hence    the studies will be excluded if only per    challenge results are reported.

### Study selection

The titles will be checked independently by two reviewers according to the above selection criteria and categorised as: included, excluded or unsure. For those papers in the unsure category, we will retrieve the abstract and re-evaluate using the same categorisation. Any discrepancies will be resolved by consensus and, if necessary, a third reviewer will be consulted. Full text copies of potentially relevant studies will be obtained and their eligibility for inclusion will be independently assessed. Studies that do not fulfil all of the inclusion criteria will be excluded.

### Data extraction and management

Data will be independently extracted onto a customised form by two reviewers, and any discrepancies will be resolved by discussion or, if agreement cannot be reached, by arbitration by a third reviewer.

Study characteristics will be collected and the number of true positives (TP), true negatives (TN), false positives (FP), and false negatives (FN) will be recorded for constructing a 2x2 table for each study. If the 2x2 data are not available, attempts will be made to derive them from reported summary statistics such as sensitivity, specificity and/or likelihood ratios.

### Quality assessment strategy

Quality assessments will be performed using the QUADAS-2 tool [5]. Each study will be independently assessed by two reviewers. Any discrepancies will be resolved by discussion or, if agreement cannot be reached, by arbitration by a third reviewer.

We will assess the quality of evidence for diagnosis, rating the evidence for each test as:

•High: Further research is very unlikely to change our confidence in the estimates of test performance.

•Moderate: Further research is likely to have an important impact on our confidence in the estimates of test performance and may change the estimates.

•Low: Further research is very likely to have an important impact on our confidence in the estimates of test performance and is likely to change the estimates.

### Data analysis and synthesis

We will examine test accuracy according to food allergy type, food allergen studied and food allergy test type. Preliminary exploratory analyses will be conducted for each test by plotting estimates of sensitivity and specificity from each study on forest plots and in receiver operating characteristic (ROC) space. These analyses will enable visual assessment of the variation between studies, and will also facilitate subgroup analyses for exploring the effect of certain characteristics on test performance.

Because we anticipate that the cut-offs used to define test positivity may vary between studies of some tests, where appropriate, we will use the hierarchical summary ROC (HSROC) model [6,7] to summarise the accuracy of each test and to compare the accuracy of two or more tests. The HSROC model accounts for within study variability, and unexplained differences between studies through the inclusion of random effects. The model uses study-specific estimates of the true positive rate (sensitivity) and the false positive rate (1 - specificity) to estimate a SROC curve. If all the parameters of the HSROC model cannot be reliably estimated due to a limited number of studies, we will simplify the model by assuming a symmetrical shape for the curve. Where studies have used a common or similar cut-off, we will use parameter estimates from the models to compute summary sensitivities and specificities with 95% confidence regions.

For making comparisons between tests, we will initially include all studies in the analysis (indirect comparison). Subsequently, if data are available, we will restrict the analyses to only studies that have compared tests in the same population, either within patients or between randomised groups (direct comparison). Such analyses are likely to produce results not confounded by differences in study or patient characteristics.

To investigate heterogeneity, the following covariates will be added to the HSROC model to assess the association of study and patient characteristics with test performance: country, different cut-offs for the index test and whether all participants received the reference standard. Sensitivity analyses will be used to explore the effect of potentially influential studies and study quality. Preliminary analyses will be done using RevewManager 5.2 (The Nordic Cochrane Centre, The Cochrane Collaboration, 2012), and the HSROC model will be fitted using the NLMIXED procedure in the SAS software (version 9.2; SAS Institute, Cary, NC). We will calculate summary ROC curves by transforming sensitivity and specificity pairs (weighted by sample size) using logistic transforms and regressing logit sensitivities on logit specificities. Summary ROC curves will be calculated by back transforming predicted values from these regression models. We will calculate the 95% confidence interval (CI) for the summary ROC curves by re-estimating the curves on bootstrap samples.These analyses will be performed with Stata version 10.0 (StataCorp LP, College Station, Texas). A narrative synthesis of the data will also be undertaken.

### Reporting

This review has been registered with the International Prospective Register of Systematic Reviews (PROSPERO) and has the registration number CRD42013003707 allocated to it. The Preferred Reporting Items for Systematic Reviews and Meta-Analyses (PRISMA) checklist will be used to guide the reporting of the systematic review [8].

## Discussion

Our review will concentrate on four type of tests: APT, SPT, specific-IgEs, and component specific-IgEs. The decision on which tests to use in clinical practice need to be made on a range of considerations, these including the populations being cared for, the tests available, their relative safety, costs and diagnostic properties. The focus of this review will very much been on the latter consideration.

## Abbreviations

APT: Atopy patch testing; CDSR: Cochrane Database of Systematic Reviews; CI: Confidence interval; DARE: Database of Reviews of Effectiveness; DBPCFC: Double-blind, placebo-controlled food challenge; EAACI: European Academy of Allergy and Clinical Immunology; EED: Economic Evaluations Database; HSROC: Hierarchical summary receiver operating characteristic; HTA: Health Technology Assessments; OFC: Open food challenge; PROSPERO: Prospective Register of Systematic Reviews; PRISMA: Preferred Reporting Items for Systematic Reviews and Meta-Analyses; ROC: Receiver operating characteristic; SPT: Skin prick testing.

## Competing interests

The authors declare that they have no competing interests.

## Authors’ contributions

KS-W, SSP, TR and YT conceptualised and designed the protocol and drafted earlier versions of the document in their capacity as methodologists. TW, AM, KH-S and GR contributed to further refinements of the protocol and revised it critically for important intellectual content in their capacity as guideline leads. AS led on the development of concepts used in this protocol and revised it critically for important intellectual content in his capacity as the methodology lead. All authors approved the final version to be published.

## Supplementary Material

Additional file 1 Search strategies. Click here for file
