# Supplementary material for: The diagnosis of food allergy: protocol for a systematic review
Source: Clin Transl Allergy. 2013 Jun 7;3:18. doi: 10.1186/2045-7022-3-18 (PMC3679851; doi:10.1186/2045-7022-3-18)
Supplement: Additional file 1 — Search strategies. [file 2045-7022-3-18-S1.docx]

**Appendix 1: Search strategies**

*Database: Ovid MEDLINE(R) In-Process & Other Non-Indexed Citations and Ovid MEDLINE(R) <1946 to Present>*

Search Strategy:

--------------------------------------------------------------------------------

| 1 | exp Food Hypersensitivity/ |
| --- | --- |
| 2 | foodallerg*.mp. |
| 3 | food hypersensitivity.mp. |
| 4 | food hypersensitivities.mp. |
| 5 | allergy, food.mp. |
| 6 | or/1-5 |
| 7 | (rat or rats or cow or cows or chicken? or horse or horses or mice or mouse or bovine or animal$).ti,ab. |
| 8 | exp animals/ not humans.sh. |
| 9 | 7 or 8 |
| 10 | 6 not 9 |
| 11 | "Predictive Value of Tests"/ |
| 12 | Skin Tests/ |
| 13 | Patch Tests/ |
| 14 | Immunologic Tests/ |
| 15 | Radioallergosorbent Test/ |
| 16 | in vitro tests.mp. |
| 17 | Immunoassay/ |
| 18 | basophil histamine release assay.mp. |
| 19 | food challenge*.mp. |
| 20 | diagnos$.ti. |
| 21 | (diet adj2 history).tw. |
| 22 | or/11-21 |
| 23 | 10 and 22 |

*Database: Embase Classic+Embase<1947 to 2012 September 14>*

Search Strategy:

**--------------------------------------------------------------------------------**

| 1 | exp Food Hypersensitivity/ |
| --- | --- |
| 2 | foodallerg*.mp. |
| 3 | food hypersensitivity.mp. |
| 4 | food hypersensitivities.mp. |
| 5 | allergy, food.mp. |
| 6 | or/1-5 |
| 7 | (rat or rats or cow or cows or chicken? or horse or horses or mice or mouse or bovine or animal$).ti,ab. |
| 8 | exp animals/ not humans.sh. |
| 9 | 7 or 8 |
| 10 | 6 not 9 |
| 11 | "Predictive Value of Tests"/ |
| 12 | Skin Tests/ |
| 13 | Patch Tests/ |
| 14 | Immunologic Tests/ |
| 15 | Radioallergosorbent Test/ |
| 16 | in vitro tests.mp. |
| 17 | Immunoassay/ |
| 18 | basophil histamine release assay.mp. |
| 19 | food challenge*.mp. |
| 20 | diagnos$.ti. |
| 21 | (diet adj2 history).tw. |
| 22 | or/11-21 |
| 23 | 10 and 22 |

*Database: CINAHL*

Search Strategy:

| S16 | S9 and S15 |
| --- | --- |
| S15 | S11 or S12 or S13 or S14 |
| S14 | (MH "Predictive Value of Tests") |
| S13 | (MH "Diagnosis, Laboratory") |
| S12 | (MH "Diagnosis, Differential") |
| S11 | (MH "Diagnosis") |
| S9 | S1 or S8 |
| S8 | S6 and S7 |
| S7 | S4 or S5 |
| S6 | S2 or S3 |
| S5 | AB allergy or allergic or hypersensitive or hypersensitivity or sensitive or sensitivity or intolerant or intolerance or reaction |
| S4 | TI allergy or allergic or hypersensitive or hypersensitivity or sensitive or sensitivity or intolerant or intolerance or reaction |
| S3 | AB food or nutrient |
| S2 | TI food or nutrient |
| S1 | (MM "Food Hypersensitivity") |

*Database: ISI Web of Science*

Search strategies:

# 2

886

Topic=(food or nutrient) AND Topic=(allergy or allergic or hypersensitive or hypersensitivity or sensitive or sensitivity or intolerant or intolerance or reaction )

Refined by: Web of Science Categories=( ALLERGY OR IMMUNOLOGY ) AND Document Types=( PROCEEDINGS PAPER OR MEETING ABSTRACT )

Databases=CPCI-S Timespan=All Years

Lemmatization=On

# 1

6,341

Topic=(food or nutrient) AND Topic=(allergy or allergic or hypersensitive or hypersensitivity or sensitive or sensitivity or intolerant or intolerance or reaction )

Databases=CPCI-S Timespan=All Years

Lemmatization=On

*Database: Cochrane Library*

Search strategy:

#1 (food hypersensitivity or (food* and (allergy or allergies or allergic or allergen*))):ti,ab,kw (Word variations have been searched)

#2 (diagnosis or diagnoses or diagnose or diagnostic* or diagnosing or test or tests or testing)

#3 #1 and #2

*Database: TRIP Database*

Search Stategy: (Advanced search screen)

area:"Allergies and Immunology"

any of these words: food allergydiagnos*

Downloaded: Evidence Based Synopses, Systematic Reviews, Guidelines

Start year (inclusive): all years

End year (inclusive): all years

*Database: Clinicaltrials.gov*

Search Strategy: (Advanced search screen)

Conditions: food allergy diagnosis

(all years)
